# The influence of rest break frequency and duration on physical performance and psychophysiological responses: a mining simulation study

**DOI:** 10.1007/s00421-022-04979-3

**Published:** 2022-06-21

**Authors:** Kanon Uchiyama, James King, Karen Wallman, Sarah Taggart, Cory Dugan, Olivier Girard

**Affiliations:** School of Human Sciences (Sport Science, Exercise and Health), The University of Western Australia, Perth, Australia

**Keywords:** Mining industry, Rest breaks, Occupational heat stress, Cognitive ability, Dehydration

## Abstract

**Purpose:**

To investigate the influence of shorter, more frequent rest breaks during simulated work (outdoor mining) in the heat on physical performance and psychophysiological responses.

**Methods:**

On separate days, thirteen males undertook two 225 min simulation trials in the afternoon (12.00–3.45 pm) including 180 min of treadmill walking at a constant rate of perceived exertion of 11 (or ‘light’) on the 6–20 Borg scale in a heat chamber (37 °C, 40% RH), interspersed with 45 min of rest breaks in an air-conditioned room (22 °C, 35% RH). Rest breaks in the current practice (CP) trial occurred at 1.00 and 2.30 pm (30 min and 15 min, respectively), while in the experimental (EXP) trial were at 1.00 (15 min), 1.45, 2.25 and 3.05 pm (10 min each).

**Results:**

Total distance covered was not different (*p* = 0.086) between CP (12,858 ± 2207 m) and EXP (12,094 ± 2174 m). Heart rate, thermal sensation and thermal comfort were significantly higher at 120–180 min (all *p* < 0.05) in CP compared to EXP. Moderate- to large-effect sizes (Hedge’s g) between trials were also found at 120–180 min for core temperature (*g* = 0.50 and 0.99, respectively). No differences were found between trials for cognitive performance, perceived fatigue, urine specific gravity, or total water intake (*p* > 0.05).

**Conclusion:**

Shorter, more frequent rest breaks have little impact on physical performance, thermal strain and exercise-related sensations. Current practices should remain in place until further studies can be conducted on an actual mine site during summer where outdoor workers perform their work duties.

## Introduction

In the North-west of Australia, many outdoor mining workers typically work ~ 11 h daily shifts over 14 consecutive days in hot ambient conditions. Performing physically demanding tasks under these circumstances can lead to hyperthermia (core temperature; T_c_ ≥ 38.5 °C). Outdoor mining workers are also obliged to wear protective clothing that covers most of their body, and includes the wearing of a hat, heavy boots and sometimes gloves. While providing protection against solar radiation, this clothing can impair the body’s ability to dissipate heat via sweat evaporation. A concomitant increase in thermal discomfort can result in workers reducing their work rate or even terminating work prematurely (Flouris et al. 2018).

Dehydration may occur if water intake is not adequate, with a 1% increase in dehydration likely to increase T_c_ by 0.1–0.2 °C (Sawka et al. 1984). Similar to hyperthermia, dehydration can impair physical performance and some complex cognitive tasks (Gopinathan et al. 1988), as well as result in heat illnesses. Even minimally dehydrated mine workers are ~ 1.5 times more likely to develop heat-related illnesses (Hunt et al. 2013). It is therefore imperative to implement strategies that aim to negate the effects of heat on outdoor mining workers so to protect their health and safety, as well as to preserve their productivity.

Studies assessing the efficacy of interventions designed to minimise the deleterious consequences of heat stress in outdoor mine workers are scarce (Ioannou et al. 2021). Of relevance to outdoor mine workers, one study reported that ice slurry ingestion extended workers’ time-to-exhaustion during a treadmill walking task in a heat chamber (~ 28 °C, ~ 74% RH) compared to water replacement (Maté et al. 2016).

Another heat-mitigation strategy is the modification of work–rest schedules where more frequent, albeit shorter rest breaks are taken over the course of a shift in a cool air-conditioned room. Short exposure (~ 10 min) to a cooler environment can reduce skin temperature (T_sk_) and T_c_, cardiovascular strain and thermal discomfort. This premise is in part supported by Zhai et al. (2019) who reported that heart rate (HR) and skin blood flow return to baseline levels within < 8 min of sitting in a thermo-neutral environment (26 °C, 50% RH), following exercise in hot environmental conditions (30 °C, 50% RH). Implementing shorter, yet more frequent rest breaks may provide more opportunity to drink refrigerated water (during rest), which would preserve hydration status as well as reduce T_c_ (Maté et al. 2016; Tippet et al. 2011). However, the effect that this practice may have on subsequent improvements in physical performance, cognitive and perceptual measures when resuming physical activity is unknown.

This mining simulation study aimed to determine the influence of manipulating rest breaks during an afternoon shift of simulated work (treadmill walking) in hot conditions on physical and psychophysiological responses. It was hypothesised that more frequent (albeit shorter) rest breaks, where overall break time was the same as current industry practices, would i) increase work done, ii) preserve cognitive function and iii) improve exercise-related sensations.

## Methods

### Participants

A repeated measures ANOVA power calculation (*α* = 0.05, 1–*β* = 0.8) was conducted with G*Power (Version 3.1.9.3) to determine a sample size based on our primary variable: physical performance (total distance covered). Based on the existing literature, the average effect size (ES) for a difference in physical performance, derived from prolonged self-pacing running laboratory studies is 0.47 (Skein et al. 2012; Wright & Davison, 2013). To express our results with 95% confidence, a minimum sample size of 12 participants was calculated for this study. Therefore, to account for dropout, thirteen male participants were recruited from the general population (mean ± SD; age: 39.3 ± 15.3 y; height: 181.5 ± 4.4 cm; body mass: 78.7 ± 11.1 kg; body mass index: 23.9 ± 3.2). All participants provided informed consent and ethics approval was granted by the host institution’s human research ethics committee (RA/4/20/6536). Testing was performed during the winter months so that participants were not heat acclimatised.

### Experimental design

**Fig. 1:**
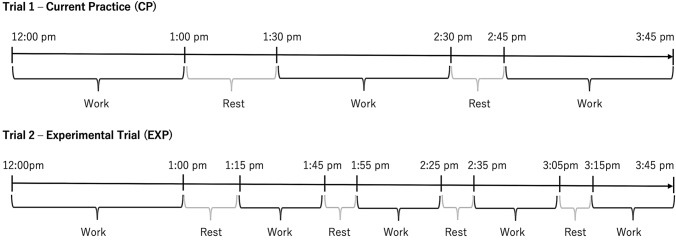
Timeline of work–rest schedule in the current practice (CP) and experimental (EXP) trials

This study used a counterbalanced, randomised, cross-over design. Participants visited the laboratory on three separate occasions: a familiarisation session, followed by two experimental trials scheduled 10 ± 8 days apart (no less than 5 days in-between). Of the two trials performed (Fig. 1), one involved usual work-to-rest break ratios undertaken by employees on a mine site in the North-west of Australia (current practice: CP trial), while the second trial had the same overall exercise and rest break duration, but rest breaks were shorter and more frequent (experimental protocol: EXP trial). Total duration of each trial was 225 min, and consisted of 180 min of simulated work, separated by 45 min of rest breaks. Simulated work was performed in a climate chamber, where the ambient environment replicated summer air temperature and relative humidity experienced on mine sites located in the North-west region of Australia (37 °C, 40% RH, 0 ms^−1^ wind speed, 0 Wm^−2^ solar radiation). Rest breaks were taken in an air-conditioned room (22℃, 35% RH, 0 Wm^−2^ solar radiation) to simulate the indoor environments provided on a mine site. All laboratory visits were performed at the exact same time of day. Physical activity (Craig et al. 2003; Hagströmer 2006; O'Brien-Cousins 1994), food and fluid intake were recorded in diaries during the 24 h period prior to the first trial and replicated prior to the second trial. Participants brought in their own lunch during their rest break, which was weighed and replicated for the subsequent trial. Finally, particular attention was given to familiarise participants with perceptual scales.

### Familiarisation session

During a preliminary visit, participants completed the Exercise & Sports Science Australia pre-exercise screening tool to identify any contraindications for physical activity. This was then followed by anthropometric measurements (body mass, height, waist and hip girth). Participants were fitted for the clothing to be worn during the trials and then practiced the counting span task (see below), which they performed five times to minimise any learning effects (Saldaris et al. 2020). Participants then completed 5 min of treadmill walking at various ratings of perceived exertion (RPE: ranging between 9 and 13) in the climate chamber (~ 27 °C, ~ 24% RH) for habituation.

### Experimental trials

Simulated work involved walking on a treadmill at a perceptually regulated intensity that equated to a RPE of 11 (or ‘light’) on the 6–20 Borg scale (Borg 1982). This corresponded to the average RPE determined from thirteen outdoor workers (with comparable anthropometrics to our participants), whilst working on a mine site in the North-west of Australia in the afternoon during the summer months (37 °C, 40% RH, 0 ms^−1^ wind speed, 0 Wm^−2^ solar radiation: unpublished observations). Walking was chosen as it represents a common activity performed by all outdoor workers across all trades (e.g. gardeners, grounds staff and tradesmen). Participants wore standard mining personal protective equipment (long-sleeve industrial shirt and pants with steel-cap boots and hat) which equated to a clothing insulation value of > 0.78 clo (American Society of Heating Refrigerating and Air-Conditioning Engineers [ASHRAE], 2013). Participants were assessed before, during and after the simulation in both trials in respect to physical and cognitive performance, perceived fatigue, physiological and perceptual parameters and hydration status.

Upon arrival, baseline measures (nude body mass, perceived fatigue, thermal sensation [TS] and thermal comfort [TC], HR, T_c_ and T_sk_, and urine specific gravity [USG]) were recorded. After entering the chamber, cognitive function was assessed before participants commenced walking. They were reminded then and every 10 min thereafter, to adjust treadmill speed at any time so to match their current work rate to an RPE of 11. Simulated work was terminated when: i) the protocol was completed (*n* = 23), ii) if T_c_ reached 40 °C (*n* = 0), or iii) if volitional exhaustion occurred (*n* = 3). If participants decided to stop walking due to exhaustion (whilst maintaining an RPE of 11), they were given the opportunity to recommence walking when they felt ready or to terminate the session. Whilst walking in the heat chamber, participants could drink room temperature water (~ 37 °C) ad libitum and had unlimited access to refrigerated water (5 °C) during rest breaks, with all water intake recorded. Urine output was also measured during toilet breaks using a labelled container. Various measures were taken during the protocol (details provided below), while all measurements were recorded again on completion of the protocol.

### Measurements

Treadmill distance (m) was used to assess physical performance and was recorded via a computerised program (Treadmill Measuring System Version 2.0, UWA, Australia) with data averaged every 30 min. The treadmill display board (speed and distance) was hidden from participant viewing to ensure condition blinding.

Six hours prior to arrival, participants ingested a radiotelemetry pill to measure gastrointestinal T_c_ (CorTemp, HQ Inc, USA). On arrival and at the end of the protocol, a mid-stream urine sample was collected to determine USG (Kavouras, 2002), using a desktop refractometer (Model T3-NE, Atago Co. Ltd., Japan) to establish hydration status. Nude body mass was then measured to the nearest 0.05 kg using a digital platform (Model ED3300; Sauter Multi-Range Ebingen, Germany); this was also repeated immediately after the protocol (towel dried) for the calculation of sweat loss (pre – post nude body mass + food + water intake + urinary output). A HR monitor (Polar RS400, Finland) was fitted around the participant’s chest, and thermistors (Skin Sensor SST-1, Physitemp Instruments Inc, NJ, USA) were taped to three sites: sternum, right medial forearm, and right mid-posterior calf to determine mean T_sk_ via a computerised program (DASYLab Light, National Instruments, Ireland Resources Ltd.). Mean T_sk_ was calculated using the formula of Burton (1935): T_sk_ = (0.5 × sternum temperature) + (0.14 × forearm temperature) + (0.36 × calf temperature). Measures of TS and TC were obtained every 5 min during the protocol on visual analog scales ranging from green to red scale (0 = ‘very cold’ to 20 = ‘very hot’) and from white to black graduation (0 = ‘very comfortable’ to 20 = ‘very uncomfortable’)*,* respectively (Gaoua et al. 2012). These physiological (T_c_, T_sk_ and HR) and perceptual (TS and TC) measures were obtained at baseline and every 5 min thereafter, with the exception of T_sk_ which was not recorded during rest breaks.

Short-term working memory was assessed using the counting span task (Case et al. 1982; Millisecond Software, Seattle, USA), with assessments made upon entering the climate chamber and after every 60 min of simulated work. Briefly, participants were presented with a number of cards (‘spans’) which included yellow and green dots. They were required to remember the number of green dots presented on each card in the order of presentation within the span and then to recall this information later. Counting span performance was recorded as the highest level in which participants successfully recalled the spans, plus one-third of a point for each span recalled at a higher level (Case et al. 1982).

Perceived fatigue was assessed before and after the treadmill exercise using the Multidimensional Fatigue Inventory (Smets et al. 1995). This questionnaire measures the sub-dimensions of general, mental and physical fatigue, as well as reduced motivation and reduced activity. The Multidimensional Fatigue Inventory has displayed good internal consistency between general, physical and mental fatigue sub-dimensions (Cronbach’s alpha > 0.80) and adequate reliability for reduced activity and reduced motivation sub-dimensions (Cronbach’s alpha > 0.65) (Lin et al. 2009). Test–retest reliability had also been reported to be between 0.59 and 0.68 for each of the sub-dimensions of perceived fatigue (Chung et al. 2014).

### Statistical analysis

Data are expressed as mean ± SD. Statistical analysis was conducted using IBM SPSS Statistics (Version 24.0 for Windows; SPSS Inc, Chicago, IL). Two-way repeated measures ANOVAs (time and condition) were used to compare distance covered, USG, fluid intake, T_c_, T_sk_, HR, TS, TC, cognitive function and perceived fatigue. One-way repeated measures ANOVAs were used to compare sweat loss between trials. All main effects were assessed using Cohen’s d (Cohen, 1988), derived from the partial eta and eta^2^ to minimise publication of error in ES (Levine & Hewett, 2002). Where appropriate, post hoc comparisons using Bonferroni adjustments were conducted. Due to our sample size (*n* < 20), Hedge’s *g* ES were assessed to determine meaningful differences, with only moderate (*g* = 0.38–0.75) and large ES (*g* ≥ 0.76) reported (Brydges, 2019; Cumming, 2012). Mean difference ± 95% confidence intervals were also determined. Statistical significance was accepted at *p* ≤ 0.05.

## Results

### Physical performance (*Fig. **2*)

**Fig. 2:**
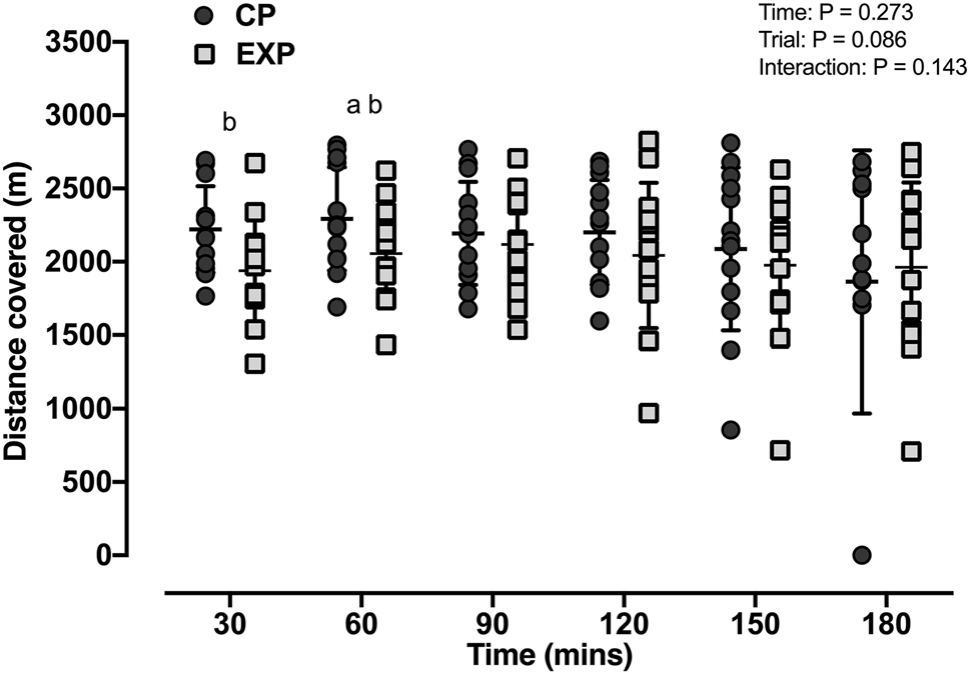
Distance covered by participants in each 30 min interval of simulated work in current practice (CP) and experimental (EXP) trials (*n* = 13). *a* = *moderate-*effect size between 30 and 60 min for EXP (*g* = 0.51 [–0.07, 1.06]). *b* = *moderate-*effect sizes between trials (*g* = 0.49 [–0.07, 1.02] to 0.68 [0.08, 1.26])

While there were no significant main effects of time (*p* = 0.273) and condition (*p* = 0.086) or an interaction effect between these factors (*p* = 0.143) for treadmill distance, moderate ES were found between trials at 30 min (CP: 2221 ± 295 m; EXP: 1938 ± 350 m; *g* = 0.68 [0.08,1.26]) and 60 min (CP: 2292 ± 352 m; EXP: 2055 ± 90 m; *g* = 0.59 [0.01, 1.16]; Fig. 2). A moderate ES was also found in total accumulative distance after 3 h of simulated work (CP: 12,858 ± 2207 m; 12,094 ± 2174 m; *g* = 0.49 [–0.07, 1.02]).

### Hydration status

#### Water intake

Total water intake did not differ (*p* = 0.993) between CP (3610 ± 1631 ml) and EXP (3612 ± 153 ml). Significantly more cold water was consumed during rest breaks in EXP compared to CP (1052 ± 605 versus 702 ± 425 ml; *p* < 0.01, *g* = 0.83 [0.20, 1.44]), while water intake was less (albeit not significantly) during work (CP: 2908 ± 1787 ml; EXP: 2561 ± 1157 ml; *p* = 0.19, *g* = 0.37 [–0.18, 0.91]).

#### Urine specific gravity

In respect to USG, there were no significant main effects for time (*p* = 0.447) or condition (*p* = 0.797), nor was there an interaction effect (*p* = 0.462). Values measured before (1.015 ± 0.011 and 1.016 ± 0.011, respectively) and after (1.013 ± 0.010 and 1.013 ± 0.014, respectively) for both CP and EXP categorised participants as being ‘minimally dehydrated’ throughout the protocol (Kavouras, 2002).

#### Sweat loss

With consideration for change in nude body mass (CP: + 1.15 ± 2.02 kg; EXP: + 1.30 ± 1.32 kg), food/water intake (CP: 3673 ± 1,601 g; EXP: 3365 ± 1037 g) and urinary output (CP: 222 ± 301 ml; EXP: 326 ± 301 ml), total sweat loss did not significantly differ between trials on completion of the protocol (CP: 2523 ± 984 ml, EXP: 2368 ± 755 ml; *p* = 0.194).

### Physiological and perceptual variables *(*Table 1)

**Table 1:** Physiological and perceptual responses to simulated work (*n* = 13)

Variables	Time (mins)							ANOVA *p*-value		
								(Cohen’s d)		
	0	30	60	90	120	150	180	Time	Trial	Interaction
Physiological variables										
Core temperature (°C)										
CP	37.20 ± 0.20	37.37 ± 0.19^*a^	37.84 ± 0.26^*§ab^	37.74 ± 0.19^*abc^	38.11 ± 0.21^*§abc^	37.93 ± 0.12^*§ab^	38.29 ± 0.21^*§abc^	**<** 0.001	0.106	0.167
EXP	37.12 ± 0.43	37.27 ± 0.32^*a^	37.76 ± 0.28^*§ab^	37.44 ± 0.63^*ab^	37.83 ± 0.34^*a^	37.87 ± 0.32^*a^	37.90 ± 0.40^*a^	(1.60)	(0.48)	(0.40)
Skin temperature (°C)										
CP	35.50 ± 0.77^c^	36.06 ± 0.35^*ac^	36.40 ± 0.51^*ab^	35.06 ± 0.72^§†bc^	36.27 ± 0.56^*§†abc^	35.35 ± 0.73^§†bc^	36.12 ± 1.01^§ab^	0.008	0.750	0.005
EXP	35.19 ± 0.56	35.91 ± 0.45^*a^	36.55 ± 0.39^*§ab^	35.75 ± 0.47^*§ab^	35.80 ± 0.68^*a^	36.83 ± 0.56^*a^	36.08 ± 0.48^*§ab^	(1.46)	(0.08)	(0.63)
Heart rate (bpm)										
CP	87 ± 10	97 ± 13^*a^	110 ± 16^*§ab^	103 ± 15^*§abc^	117 ± 18^*§abc^	108 ± 16^*§ab^	122 ± 20^*§†abc^	< 0.001	0.589	< 0.001
EXP	88 ± 13	99 ± 14^*a^	113 ± 17^*§ab^	110 ± 19^*ab^	113 ± 22^*ab^	113 ± 24^*a^	116 ± 24^*§ab^	(2.11)	(0.11)	(0.36)
Perceptual variables										
Thermal sensation										
CP	13 ± 2^†c^	14 ± 2^ac^	15 ± 2^*§abc^	13 ± 2^§b^	16 ± 2^*§†abc^	13 ± 2^§b^	15 ± 3^*§ab^	0.006	0.456	< 0.001
EXP	14 ± 2	15 ± 2^a^		14 ± 2^§b^	14 ± 2^§b^	14 ± 1	15 ± 1^§b^	(0.99)	(0.19)	(0.72)
Thermal comfort										
CP	10 ± 4	12 ± 3^*a^	14 ± 2^*§ab^	12 ± 2^§abc^	15 ± 2^*§abc^	12 ± 3^§ab^	15 ± 3^*§†abc^	0.002	0.985	0.008
EXP	11 ± 4	13 ± 2^a^	14 ± 2^*§ab^	13 ± 1^§ab^	13 ± 1^ab^	13 ± 2^a^	13 ± 2^*ab^	(1.25)	(0.09)	(0.50)

Mean ± SD. The simulated work protocol required participants to walk for a total of 180 min with work–rest schedules reflecting the current practice (CP) and the experimental trial (EXP)

^*^significantly different from 0 min (*p* < 0.05)

^§^significantly different from previous time-point (*p* < 0.05)

^†^significantly different between CP and EXP trials (*p* < 0.05)

^a^moderate- to large-effect sizes between time points, compared to 0 min (*g* = 0.40 [–0.15, 0.95] to 3.05 [1.60, 4.47])

^b^moderate- to large-effect sizes between time points, compared to the previous time point (*g* = 0.42 [–0.14, 0.97] to 4.06 [2.12, 5.99])

^c^moderate- to large-effect sizes between CP and EXP trials (*g* = 0.42 [–0.22, 1.03] to 1.33 [0.42, 2.20])

#### Core temperature

There was a significant main effect for time (*p* < 0.001), but not for condition or time × condition interaction (Table 1). Pooled values for CP and EXP showed significant differences for all time points in reference to baseline (*p* < 0.05), as well as significant differences between 30 and 60 min, and 150 and 180 min (*p* < 0.05). Over the course of the protocol, T_c_ increased by 1.00 ± 0.39 °C (*g* = 2.45 [1.24, 3.64]) and 0.78 ± 0.55 °C (*g* = 1.37 [0.49, 2.21]) in CP and EXP, respectively.

#### Skin temperature

While there was no main effect for condition, there were significant time (*p* = 0.008) and time × condition interaction (p = 0.005) effects (Table 1). Compared to CP, T_sk_ was elevated by 0.68 ± 0.49 °C (*p* < 0.05, *g* = 1.33 [0.42, 2.20]) after 90 min and by 0.48 ± 0.53 °C (*p* < 0.05, *g* = 0.87 [0.10, 1.60]) after 150 min in EXP. Conversely, T_sk_ was elevated by 0.31 ± 0.55 °C (*g* = 0.54 [–0.15, 1.20]*)* at baseline, by 0.15 ± 0.27 °C (*g* = 0.52 [–0.17, 1.17]) after 30 min, and by 0.48 ± 0.59 °C (*p* < 0.05, *g* = 0.77 [0.03, 1.55]) after 120 min in CP compared to EXP. Over the course of the protocol, T_sk_ significantly increased by 1.10 ± 0.94 °C (*p* < 0.05; *g* = 1.12 [0.19, 1.54]) and by 0.89 ± 1.73 °C (*g* = 0.49 [–0.15, 1.12]) in EXP and CP, respectively.

#### Heart rate

There was a main effect for time and a time × condition interaction for HR (both *p* < 0.001; Table 1). Compared to EXP, HR was higher in CP by 7 ± 13 bpm at 120 min (*g* = 0.57 [–0.07, 1.22]) and by 9 ± 10 bpm at 180 min (*p* < 0.05, *g* = 0.89 [0.19, 1.56]). At 90 min, HR was higher in EXP by 5 ± 10 bpm, comapred to CP (*g* = 0.46 [–0.16, 1.05]). All time points in both trials were significantly different from their respective baseline values (*p* < 0.05, *g* = 0.79 [0.11, 1.44] to 1.98 [1.02, 2.91]). Over the course of the protocol, HR significantly increased by 35 ± 20 bpm in CP (*p* < 0.05, *g* = 1.70 [0.76, 2.61) and by 27 ± 20 bpm in EXP (*p* < 0.05, *g* = 1.28 [0.53, 1.91).

#### Thermal sensation

There was a main effect for time (*p* = 0.006) and a time × condition interaction (*p* < 0.001) for TS. Compared to EXP, TS was significantly higher (feeling hotter) in CP at 120 min (*p* < 0.05, *g* = 0.77 [0.10, 1.47]). Conversely, TS was signficantly higher in EXP at the commencement of simulated work (*p* < 0.05; *g* = 0.95 [0.23, 1.63]), 30 min (*g* = 0.58 [–0.05, 1.19]) and 60 min (*g* = 0.41 [–0.20, 0.99]).

#### Thermal comfort

The main effect for time (*p* = 0.002) and the time × condition interaction (*p* = 0.008) were significant for TC. Between trials, scores were higher (less comfortable) for CP at 120 (*g* = 0.54 [–0.09, 1.14]) and 180 min (*p* < 0.05, *g* = 0.73 [0.07, 1.37]), with scores being higher at 90 min in EXP (*g* = 0.52 [–0.11, 1.11]).

#### Cognitive performance (*Fig. **3*).

**Fig. 3:**
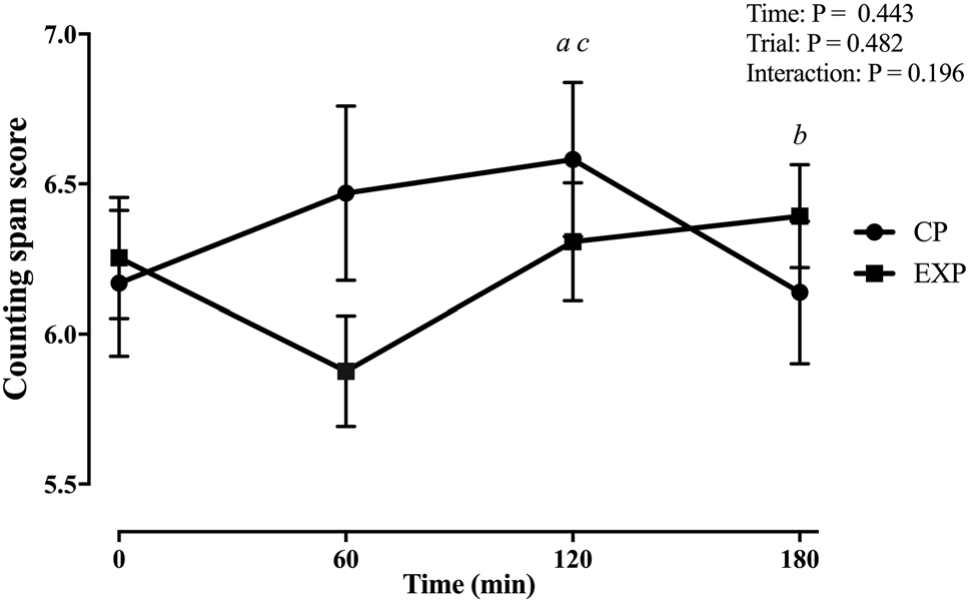
Counting span scores (mean ± SEM) for the current practice (CP) and experimental (EXP) trials (*n* = 13). *a* = moderate-effect size between 0 and 120 min for CP (*g* = 0.54 [–0.04, 1.10]). *b* = moderate-effect size between 120 and 180 min for CP (*g* = 0.72 [0.09, 1.32]). *c* = moderate-effect size between trials (*g* = 0.40 [–0.18, 0.96])

There were no significant main effects of time (*p* = 0.443) and condition (*p* = 0.482) or a significant interaction between these factors (*p* = 0.196) for the counting span task.

#### Perceived fatigue (*Fig. **4*).

**Fig. 4:**
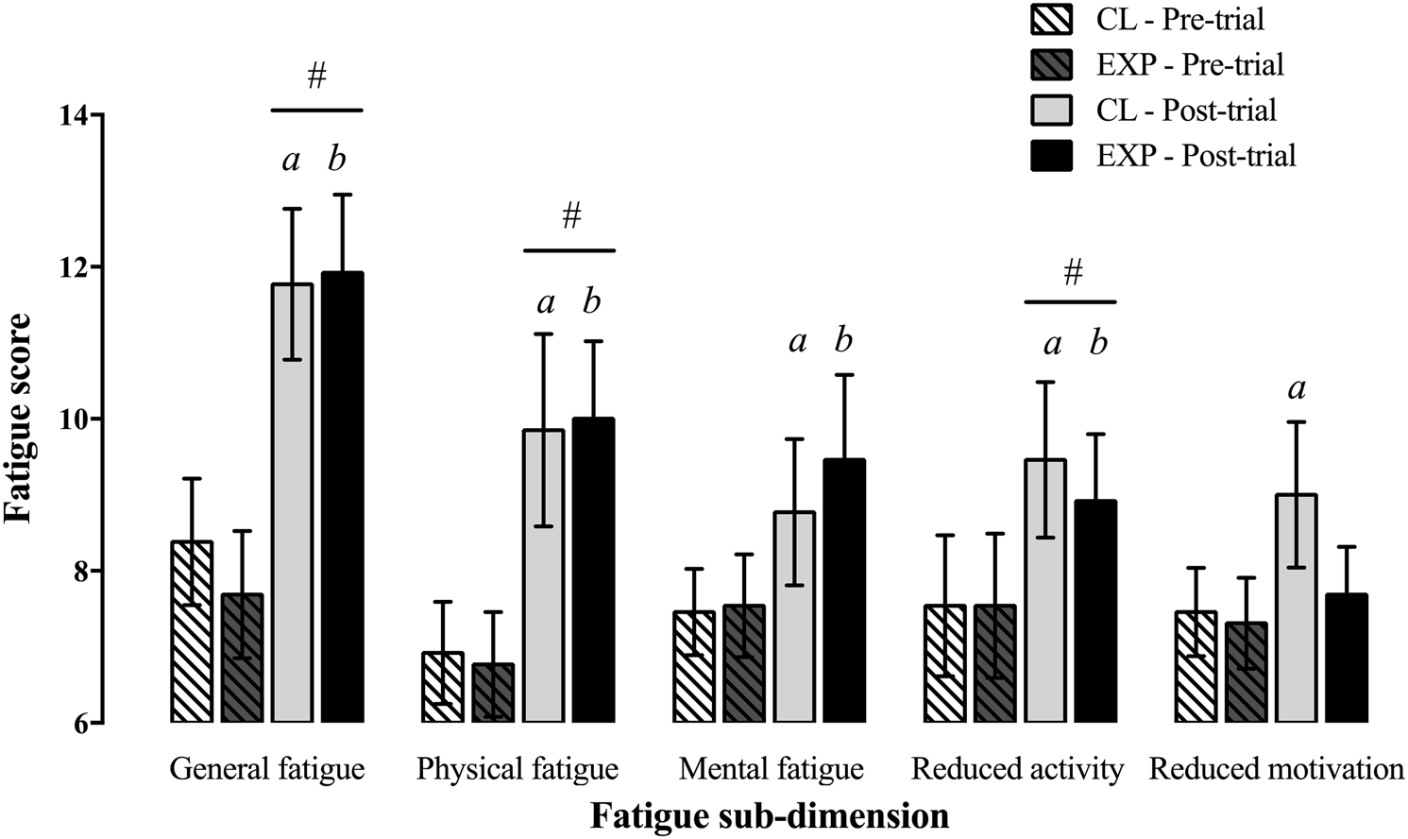
Sub-dimensions of perceived fatigue (mean ± SEM) for current practice (CP) and experimental (EXP) trials (*n* = 13). *#* = significant difference (*p* < 0.05) for post-trial relative to pre-trial, globally. *a* = moderate- to large-effect size (*g* = 0.39 [–0.17, 0.93] to 1.39 [0.61, 2.13]) between pre- and post-trial for CP. *b* = moderate- to large-effect size (*g* = 0.66 [0.06, 1.23] to 1.29 [0.55, 2.01]) between pre- and post-trial for EXP

Scores for general (+ 55.1 ± 48.3%, *p* < 0.001), physical (+ 44.0 ± 39.8%, *p* = 0.001) and mental fatigue (+ 20.8 ± 33.5%, *p* = 0.058), as well as reduced activity (+ 30.5 ± 46.9%, *p* = 0.020) and reduced motivation (+ 13.1 ± 21.7%, *p* = 0.063) increased globally from before to after the exercise protocol. Neither the time × condition interaction (all *p* > 0.203) nor the main effect of condition (all *p* > 0.186) reached statistical significance for any of the five sub-dimensions of perceived fatigue.

## Discussion

### Physical performance

Physical performance (treadmill distance) did not differ significantly between trials. This contradicts our hypothesis that shorter, more frequent rest breaks would subsequently improve exercise capacity in the heat. However, these observations are in line with a field-based study by Sawka et al. (2015) reporting no differences between continuous and intermittent (work–rest ratio: 30–10 min) military pacing at a moderate intensity (~ 400 W) in the heat (~ 29 °C wet bulb globe temperature). It is possible that circulatory adjustments incurred via the transition from rest to work may have disrupted physical performance in EXP. During exercise in the heat, the demand for blood flow to the periphery (for cooling) is contested by the working muscles (for movement) (Périard et al. 2021). During rest, however, participants opted to be seated, thereby reducing the demand for skeletal muscle pump activity (Crandall and Wilson, 2015). Consequently, blood flow during rest breaks would have been displaced from the working muscles (legs) and redirected from the working muscles (legs) to the periphery (skin) for heat dissipation (Johnson and Rowell, 1975). Blood flow redistribution may have accentuated venous pooling (in the legs), which would have consequently reduced venous return and cardiac filling when commencing subsequent work-intervals (Périard et al. 2021). With RPE strongly determined by cardiovascular redistribution (Périard et al. 2011), this may have increased participants’ perception of work at the commencement of each work-interval (following rest breaks), influencing their adjustment of intensity (RPE of 11). Therefore, the intervention of additional rest breaks in EXP compared to CP may have induced circulatory adjustments at the resumption of each subsequent work interval (following rest breaks), and thus disrupted physical performance.

### Hydration status

It was expected that shorter, more frequent rest breaks would encourage water ingestion (Pryor et al. 2018), in turn leading to better hydration status in EXP compared to CP. Surprisingly, urine specific gravity was stable across conditions and from before to after the exercise protocol. Conversely, Greenleaf and Sargent (1965) reported that individuals failed to drink enough water ad libitum to compensate for sweat losses, when walking (at 6.4 kmh^−1^) for 4 h in extreme heat (49 °C, 25% RH). Hydrated individuals are able to tolerate higher increases in T_c_ under heat stress, compared to dehydrated (as they experience increased cardiovascular strain; Périard et al. 2021). As many participants were either physically active and/or had a background in exercise sciences, much of the sample was likely well informed of the importance of fluid ingestion and the potential detrimental impacts of dehydration. These individuals may have consumed water in excess to overcompensate for fluid loss and prevent dehydration. Altogether, results showed that participants were able to self-manage in both trials, which may have aided in their physiological tolerance to heat stress and maintenance of physical performance throughout the exercise protocol.

Importantly, water intake inside the climate chamber (during work) in both trials may have been greater than anticipated due to the monotony of simulated work. While participants were permitted to have entertainment (i.e. music, television shows, podcasts; controlled between trials) inside the climate chamber, anecdotal evidence suggests boredom may have prompted them to drink. Water was available ad libitum throughout work and rest breaks, resulting in a total water consumption of ~ 3.6 L in both trials. Total water intake contributed to an increase in nude body mass of ~ 1.5% in CP and ~ 1.6% in EXP. Water consumption during CP and EXP may have also prevented the deterioration of complex cognitive performance (discussed below). It is unclear whether these findings would be consistent if replicated in a field-based study, whereby participants will be required to perform occupational tasks, often in the company of fellow workers. This may provide a distraction from hydration, as work tasks involve increased stimuli and decision-making, compared to treadmill walking. Therefore, actual labour tasks may lower water ingestion during a prolonged shift, in line with field-based evidence (Hunt et al. 2013).

### Physiological and perceptual responses

The T_c_ and HR responses observed in CP and EXP concur with previous observations (Zhai et al. 2019) in that rest breaks in cooler environments can lower cardiovascular strain incurred from prolonged physical activity in the heat. This may have increased heat dissipation capacity via the attainment of a larger gradient of ambient temperature and water vapour pressure between the skin and the surrounding environment (Wendt et al. 2007). Notably, the higher T_c_ and HR values observed at 120 and 180 min time points in CP compared to EXP (with moderate-to-large ES) may represent the accumulative elevation of hourly work intervals in CP. Additional rest breaks in EXP, separated into 30 min work intervals may have aided in the alleviation of thermal strain via the restoration of thermoregulation that occurs with (1) exposure to a cooler ambient environment (Wendt et al., 2007) and (2) the cessation of exercise (Kenny & McGinn, 2017).

Of importance, the EXP trial was successful in maintaining T_c_ values below 38 °C. Therefore, shorter, more frequent rest breaks may be appropriate for the protection of worker health and safety. Nonetheless, our findings also indicated that most participants were able to tolerate hyperthermic conditions (> 38 °C) without negatively impacting physical performance. Heat exhaustion resulted in the early termination of the protocol on three occasions. While two of the three cases failed to report T_c_ values (due to inability to detect the telemetric capsule), the final T_c_ of one participant was reported to be 39.09 °C at the time of termination in CP (completed 195 of the 225 min protocol). Notably, the participant completed the EXP trial without the termination of the protocol.

Better maintenance of thermal homeostasis in the EXP trial may also be attributed to the greater consumption of cold water compared to CP. Ingestion of cold water can delay the autonomic heat dissipation mechanisms activated during activity in the heat via the reduction of thermal strain on the cardiovascular system, possibly resulting in lower TS and TC values (Wilson et al. 2002). Of relevance, the EXP trial reported lower TS at 120 min, and lower TC at 120 and 180 min (similar to reductions in T_c_ and HR). This concurs with a report by Lee et al. (2018), whereby the ingestion of cold water (4 °C) lowered TS at the end of the rest break period (45 min) and throughout subsequent time-to-exhaustion exercise in the heat (cycling), compared to warm water ingestion (37 °C). Additionally, these authors reported that 900 ml of cold water lowered T_c_ by 0.5 °C, compared to warm water, during 30 min of rest. Exposure to cooler environments and the provision of refrigerated water in rest breaks, likely explained the reduction in physiological and perceptual responses reported for EXP compared to CP.

### Cognitive performance

Our results showed no impairment of complex cognitive performance in either protocol. It was anticipated that 180 min of walking in thermally stressful conditions while wearing protective personal equipment, with fewer rest periods exposed to a cool environment, would induce moderate hyperthermia (T_c_ > 38.5 °C) in CP, and hence contribute to deterioration of complex cognitive performance. In both CP (T_c_ = 38.3 °C) and EXP (T_c_ = 37.9 °C), however, T_c_ did not exceed the proposed threshold (38.5 °C) above which complex cognitive performance is impaired (Gaoua et al. 2011; Schlader et al. 2015). Additionally, EXP prevented mean T_c_ from reaching hyperthermic levels (< 38.0 °C). Our findings, in which cognitive impairment did not occur in either protocol, were supported by previous studies which assessed complex cognitive performance during hyperthermia using short-term working memory tasks. For example, Schlader et al. (2015) reported that mild hyperthermia (T_c_ = 38.0 °C) did not impair complex cognitive performance. It was speculated that rest breaks in an air-conditioned room and access to cold water (5 °C) ad libitum likely reduced T_c_. Results from Lee et al. (2008) suggest that the ingestion of 900 mL of cold fluid (4 °C) reduces T_c_ by ~ 0.5 °C relative to ingestion of 900 mL of warm fluid (37 °C). In our study, the combination of cold water consumption and exposure to thermo-neutral conditions during rest breaks likely prevented T_c_ from exceeding 38.5 °C in CP and EXP, hence averting complex cognitive impairment.

### Perceived fatigue

All sub-dimensions of perceived fatigue increased from pre- to post-exercise protocol (albeit not significantly for mental fatigue and reduced motivation), independent of condition. This contradicts our hypothesis, as we expected lower perceived fatigue scores following completion of EXP compared to CP. Although fatigue levels were not different between trials, lower physiological strain was observed in EXP relative to CP. As fatigue likely results from extended periods of physically demanding activity (Caldwell et al. 2012), it was assumed incorporation of more frequent, shorter duration rest breaks may have resulted in lower levels of fatigue. By the end of the protocol, TC, T_c_ and HR were higher in CP than in EXP, indicating greater thermal discomfort and physiological strain. However, this did not correspond to increased perceived fatigue post-trial in CP. One potential explanation is because exercise intensity was perceptually regulated (as opposed to imposed), and therefore similar between protocols, given participants were required to maintain the same RPE in both trials. Although T_c_ and HR were higher at 120 min and 180 min in CP, the alleviation of thermal strain during the lunch break (CP: 30 min; EXP: 15 min) was comparable between conditions (all *p* > 0.05). Fatigue, measured perceptually, may have been influenced by participants’ preference of trials and/or their preconceived ideas, whereby they may have believed that longer rests alleviated fatigue. Overall, our results suggest that altering work-to-rest schedules to incorporate more frequent, shorter duration rest breaks did not result in lower fatigue accumulation relative to schedules with fewer, longer duration rest breaks.

### Limitations and additional considerations

This study is not without limitations. First, our results showed that physical performance did not differ between trials over a single simulated shift. However, with rostered swings often lasting for ~ 14 days, the impact of rest break interventions on accumulative heat strain (and its consequences on physical performance) on outdoor mine workers was not assessed. While a laboratory-based study allowed for strict control of ambient environmental conditions and the removal of confounding factors, it prevents the generalisation of our findings to real-world settings. With literature suggesting that time to exhaustion occurs sooner in laboratory versus field-based studies (Sawka et al., 2015), this indirectly highlights the distinction between simulative- and naturally incurred heat stress.

Second, while participants were selected to best replicate the demographic of outdoor mine workers, volunteer recruitment may have attracted physically fit individuals who were confident in their ability to withstand thermally stressful conditions. Heat-tolerant individuals often possess inter-individual characteristics (younger, male, with higher aerobic capacity, and lower adipose body composition) that can improve their ability to maintain thermal homeostasis under heat stress (Kenny and Jay, 2013).

One additional limitation is the absence of solar radiation in our study, which is considered one of the biggest contributors to cognitive impairment (Piil et al. 2020; Ioannou et al. 2021). While we did not observe any impairment of cognitive performance over a shift, the presence of solar radiation may have influenced this response. Alternatively, the complexity of the cognitive task may have not been suitable for the tested population. Eight trials resulted in the attainment of the maximum counting span performance for all time points within a trial, while three participants achieved the maximum counting span performance across all time points in both CP and EXP.

Finally, it is likely (yet unknown) that behavioural thermoregulation strategies may differ between laboratory and field-based studies conducted in thermally stressful conditions. Field-based research has suggested that outdoor (agricultural) workers spend ~ 15% of total work shift time on irregular unprescribed rest breaks (Ioannou et al. 2017). This contrasts with our laboratory-based protocol where rest breaks were carefully timed and controlled. While the present study reported no significant differences in total water intake between protocols, real-world replication may limit workers from sufficient ingestion due to limited water accessibility (Piil et al. 2018), which may subsequently influence worker’s urine specific gravity post trial. Overall, field-based research is needed to capture the actual behavioural worker adjustments to prolonged heat stress on work productivity and water intake.

## Conclusion

Our intention was to assess the impact of shorter, more frequent rest breaks on i) physical performance, ii) thermal, physiological and perceptual responses, and iii) perceived fatigue and cognitive function, over a 225 min treadmill protocol in hot conditions. Our main findings indicate that total distance did not differ between trials, despite lower physiological strain (T_c_ and HR) when shorter, more frequent rest breaks were implemented. Irrespective of condition, completion of this mine-simulation protocol induced fatigue, while cognitive function remained unaffected with participants staying *‘*minimally dehydrated’*.* It is therefore unlikely that the intervention of shorter, more frequent rest breaks facilitates overall physical performance under heat stress over one 225 min simulated afternoon shift. Real-world investigation is warranted to evaluate the impact of work–rest practices to optimise productivity, whilst curtailing occupational heat stress.

## Abbreviations

T_c_: Core temperature
EXP: Experimental trial
CP: Current practice trial
HR: Heart rate
T_sk_: Skin temperature
TC: Thermal comfort
TS: Thermal sensation
RPE: Ratings of perceived exertion
USG: Urine specific gravity
